# A Wireless Gas Sensor Network to Monitor Indoor Environmental Quality in Schools

**DOI:** 10.3390/s18124345

**Published:** 2018-12-09

**Authors:** Alvaro Ortiz Perez, Benedikt Bierer, Louisa Scholz, Jürgen Wöllenstein, Stefan Palzer

**Affiliations:** 1 Laboratory for Gas Sensors, Department of Microsystems Engineering, University of Freiburg, Georges-Köhler-Allee 102, 79110 Freiburg, Germany; alvaro.ortiz.perez@imtek.uni-freiburg.de (A.O.P.); Benedikt.bierer@imtek.de (B.B.); louisa.scholz@imtek.de (L.S.); Juergen.Woellenstein@ipm.fraunhofer.de (J.W.); 2 Fraunhofer Institute for Physical Measurement Techniques (IPM), Heidenhofstraße 8, 79110 Freiburg, Germany; 3 Department of Computer Science, Universidad Autónoma de Madrid, Francisco Tomás y Valiente 11, 28049 Madrid, Spain

**Keywords:** miniature photoacoustic non-dispersive infrared absorption spectroscopy (NDIR) sensor, wireless gas sensor network, indoor environmental quality, thermal comfort, carbon dioxide

## Abstract

Schools are amongst the most densely occupied indoor areas and at the same time children and young adults are the most vulnerable group with respect to adverse health effects as a result of poor environmental conditions. Health, performance and well-being of pupils crucially depend on indoor environmental quality (IEQ) of which air quality and thermal comfort are central pillars. This makes the monitoring and control of environmental parameters in classes important. At the same time most school buildings do neither feature automated, intelligent heating, ventilation, and air conditioning (HVAC) systems nor suitable IEQ monitoring systems. In this contribution, we therefore investigate the capabilities of a novel wireless gas sensor network to determine carbon dioxide concentrations, along with temperature and humidity. The use of a photoacoustic detector enables the construction of long-term stable, miniaturized, LED-based non-dispersive infrared absorption spectrometers without the use of a reference channel. The data of the sensor nodes is transmitted via a Z-Wave protocol to a central gateway, which in turn sends the data to a web-based platform for online analysis. The results show that it is difficult to maintain adequate IEQ levels in class rooms even when ventilating frequently and that individual monitoring and control of rooms is necessary to combine energy savings and good IEQ.

## 1. Introduction

The amount of time people spend indoors exceeds 90% of the total [1,2,3] and as a consequence indoor environment quality (IEQ) is a major concern for health and well-being of the general population. The concept of IEQ entails indoor air quality (IAQ), thermal comfort, as well as light and noise levels inside buildings [4]. Because of their increased vulnerability children and young adults are particularly prone to the adverse effects of poor IEQ and especially schools, which usually feature a high occupant density, are focal point of a multitude of challenges. These include the accumulation of hazardous gases and particulate matter (PM), health risks via mold formation as well as the spreading of bacteria [5]. Additionally, thermal comfort is closely linked with the cognitive performance [6] and hence both educational success and health of pupils in schools critically hinges on IEQ. Past research has established the negative health effects originating from exposure to nitrogen dioxide (NO_2_), ozone (O_3_), carbon dioxide (CO_2_), carbon monoxide (CO), volatile organic compounds (VOCs) and benzene, toluene, ethylbenzene, xylenes (BTEX) in particular, radon, and PM exposure and consequently the need to monitor and control their concentrations in schools [7,8,9], so while there is a need for large scale deployment of sensor technologies to enable monitoring IEQ with high temporal and spatial resolution in schools, currently available solutions are scarce and too costly for large area installations [5]. In particular, to date all wireless gas sensor networks face a trade-off between sensor node cost and data quality because currently no suitable, low-cost technology for specific, quantitative chemical analysis is available [10]. Researchers and companies alike therefore have to revert to technologies that approximate the gaseous pollutants content instead of specifically determining the quantity of trace gas concentrations. Commercial solutions that may serve as sensor nodes include individual sensors to monitor IAQ with varying combinations of sensors for gases and particulate matter. The main obstacle is the current lack in suitable chemical analysis technologies to specifically and sensitively determine the concentration of gaseous air pollutants at low-cost, and offering long term stable detection. Past studies therefore often had to rely on logging data with low spatial and temporal resolution of several minutes [11,12,13,14] preventing real-time assessment of IEQ and limiting large scale deployment of such systems. The underlying gas sensing technologies used are often low-cost but do not allow for specific detection of trace gases. E.g., the CO_2_ concentration is oftentimes inferred from the reading of a metal-oxide based, total volatile organic compound (TVOC) sensor, even though the correlation between TVOC and CO_2_ is weak [15]. While Raman-based approaches may detect many gases simultaneously [16,17,18,19,20,21,22], techniques based on absorption spectroscopy are the most promising candidates for reliable CO_2_ detection. Using tunable diode laser spectroscopy a high degree of sensitivity and specific, quantitative detection can be achieved, albeit at high associated costs in terms of optical and computational infrastructure as well as maintenance [23,24]. Currently, so-called non-dispersive infrared absorption spectroscopy (NDIR) is the most popular tool for CO_2_ monitoring that does not require analytical grade concentration readings [25]. Commercially available sensors achieve a resolution of ±30 ppm at an optical path length of several cm. Usually thermal emitters are used as light sources and spectral filters are employed to establish a reference channel that corrects for fluctuations of the emitted light intensity using the atmospheric window around 3.95 µm and a measurement channel to probe CO_2_ absorption bands at 2.6 µm or 4.2 µm [26,27]. The number of optical components necessary, the thermal emitter and the optical path lengths involved make for a rather bulky and comparatively expensive design. Recently, we have presented a design that builds on the original URAS idea [28], i.e., using the photoacoustic effect to gauge the light intensity of those parts of the light spectrum that are resonant to the CO_2_ absorption lines. This way LED-based gas sensors that are one order of magnitude smaller than current state-of-the art NDIR setups but comparable performance may be build [29,30]. While we have also presented a method to establish a reference channel to compensate drifts in the LED’s intensity in this setup [29], our laboratory characterization results suggest the possibility to compensate intensity fluctuations using a temperature calibration. This would open up the possibility to build the most basic form an NDIR sensor consisting only of a LED, a waveguide and a detector in a miniaturized form and featuring long-term stable operation. Other, more sophisticated, photoacoustic-based approaches allow e.g., for calibration-free monitoring or drift-reduction [31,32] but for all intends and purposes of IEQ monitoring, our simple setup suffices.

In this contribution, we therefore present results achieved by this basic setup that has been integrated into a wireless sensor network and enables the deployment of sensor nodes capable of monitoring thermal comfort as well as determining the CO_2_ concentrations specifically and within the required concentration range from 400–5000 ppm. Using our photoacoustic NDIR setup we show that long term stable operation of a LED-based CO_2_ sensor without reference channel is feasible by utilizing a suitable temperature compensation scheme. This opens up the possibility to produce low-power consuming, easy to manufacture CO_2_ gas sensors that do not feature cross-sensitivities towards humidity. Based on the data we investigate the effect of different ventilation methods on the air quality, discuss the implications on building-wide CO_2_ levels, and assess the thermal comfort. Because of the small size of the sensors nodes, their wireless internet connectivity and low-cost, the system architecture may easily be adapted to different scenarios and employed on a large scale. In particular, the approach enables in-situ monitoring with high spatial and temporal resolution inside a building and to combine the data to allow for a building performance evaluation and active control of IEQ. 

## 2. Materials and Methods

Because of its outstanding importance for indoor air quality we demonstrate a gas sensor approach for CO_2_ employing a concept that may easily be adapted for further gases including NO_2_, CO, O_3_, and CO [33,34]. Evidence suggests correlations between absenteeism [11], bacterial infestation [35,36], and performance [37] with the CO_2_ levels indoors and it also allows for determining the ventilation rate. This makes the CO_2_ concentration the single most important chemical parameter to assess indoor air quality. In order to enable large scale deployment of the system at low cost and small overall size, we make use of a novel, miniature photoacoustic-based, nondispersive infrared (NDIR) setup, based on the original URAS design [28], which makes it possible to construct miniature CO_2_ sensors with high sensitivity and without cross-sensitivities towards humidity [30]. The CO_2_ sensor module features a mid-infrared LED from Hamamatsu (Hamamatsu City, Japan) as light source emitting light around 4.2 µm, an aluminum waveguide realizing an optical path of 30 mm and guiding the LED radiation to a hermetically sealed, CO_2_ filled cell containing a SPU0409HD5H-QB MEMS microphone from Knowles (Itasca, IL, USA). To excite a sound wave inside the detector, the LED is intensity modulated at 500 Hz using a rectangular current shape with 80 mA amplitude and 50% duty cycle. Since the sound wave amplitude is directly proportional to the light intensity [38], we use its magnitude to infer the CO_2_ concentration. To do this the microphone signal is first converted into a digital signal using an analog-to-digital converter (ADC) from Analog Devices (Norwood, MA, USA) with 12-bit and digital I2C interface protocol operating at 2.5k samples read out rate. The sound wave amplitude is determined using the Goertzel algorithm [39] implemented on the PSoC microcontroller. Using an apparatus to simulate real-world conditions in the lab [40], we have performed a calibration of the CO_2_ sensor reading in dry synthetic air at 1 bar pressure. We have also checked for cross-sensitivities towards humidity as well as established the correlation between ambient temperature and sensor signal [30]. To perform temperature calibration with large temperature variations we have used a climatic chamber filled with pure dry synthetic air in the temperature range from 15 °C to 50 °C. The latter calibration is then used to correct for the influence of temperature variations during field tests. To determine ambient temperature and humidity a fully calibrated SHT21 IC sensor with low power consumption from Sensirion (Staefa, Switzerland) is connected to the microcontroller using the I2C bus. The PSoC also acts as central tool to control data transmission via a Z-Wave module, sensing data on temperature, humidity, and CO_2_ concentration is send every 30 s.

**Figure 1:**
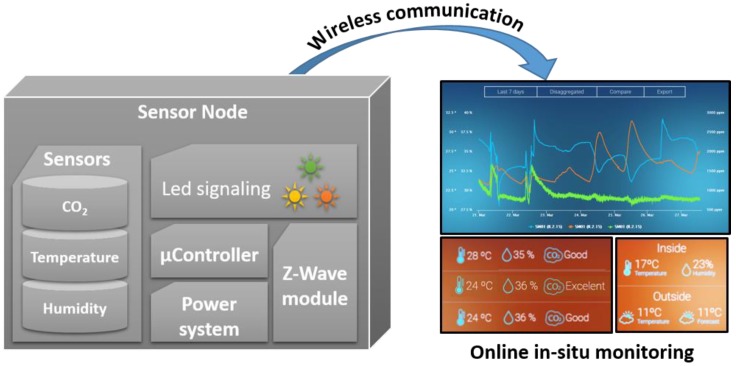
The concept of the individual sensor nodes is based on the use of micromachined sensor technology and internet connectivity in order establish a wireless network for online, in-situ indoor environmental monitoring. The CO_2_ concentration is determined by listening to its concentration via the photoacoustic effect. Both temperature and humidity are determined using state-of-the-art microtechnology.

**Figure 2:**
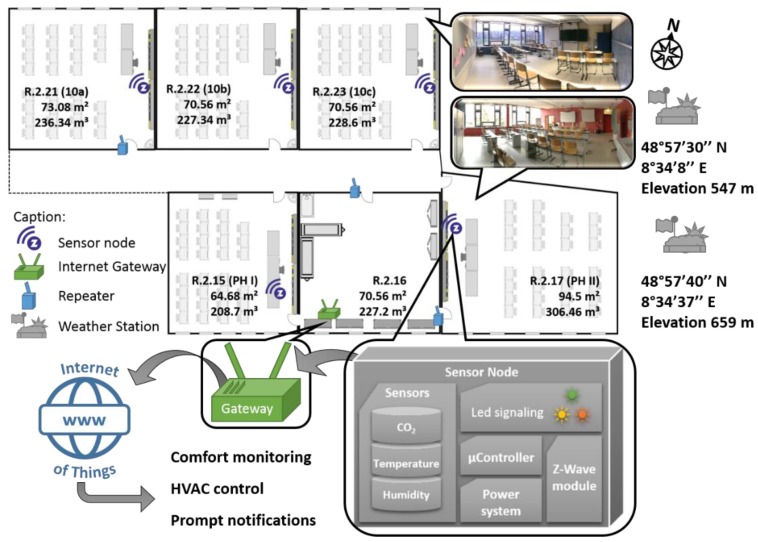
Overview of the system installation at the school. Each sensor node is installed at about 1 m height above ground. To ensure reliable data transfer three signal repeaters have been installed. Area and volume of all rooms are annotated in the schematic map and the position of each sensor node is indicated above.

The sensor node design is depicted in Figure 1 and includes wireless connectivity to make it possible to monitor the pollutant exposure of children on a micro-scale, which is needed for next generation studies of the health effects of school children [41]. At the same time the data from the whole network may be fused to offer a comprehensive picture on a building-wide level. Each node is equipped with a Z-Wave module to enable transfer of the data to an internet application by means of an internet gateway. In undisturbed environments the physical range of the transmission is about 100 m. Based on the online data platform “EnControl” from Sensing and Control Systems S.L. (Barcelona, Spain) we have created a tool to determine the thermal comfort, ventilation rate, and production rate of CO_2_ during classes. Moreover, data on thermal comfort may be used and integrated into the heating systems in order to minimize energy expenditure during winter. Using this system we investigate the result of various ventilation methods in a school as well as infer on the development of CO_2_ levels in the building as a The system has been deployed at “Gymnasium Remchingen”, a secondary school in rural Germany close to the city of Karlsruhe and the location of all sensor network components is depicted in Figure 2. The building is in operation since 2004 and currently about 470 pupils are educated in 21 classes. The building features a high level of thermal insulation and interior walls made of reinforced concrete but without active ventilation control, such that ventilation is controlled manually via opening the windows and/or class room doors. For investigating the influence of ventilation on indoor environmental quality a total of 5 rooms has been monitored each equipped with one sensor node. Because of the materials deployed in the school building the Z-Wave physical range is reduced to about 20 m, which is why a total of 3 repeaters modules has been installed for reliable signal transmission. Three of the rooms are standard class rooms and two are dedicated to physics classes. 

Each sensor module sent the current CO_2_-level, humidity as well as temperature to the cloud-based “EnControl” platform every 30 s. Outdoor conditions are monitored via two weather stations in close proximity to the school (<200 m distance) whose data is available online through the Weather Underground web portal www.wunderground.com (The Weather Company, an IBM Business). These weather stations are based on NETATMO products (www.netatmo.com).

In order to assess the air quality in the five class rooms we use the CO_2_ concentration as tracer gas. We use these values to calculate the ventilation rates (VR) as well as the background levels in each room. Among the diverse methods to calculate the air change rate (ACR) we have chosen the decay method according to VDI 4300 (2001) [42], which has been proved to be a feasible and effective way to determine the air change rates in scenarios like the ones explored in this work [43]. The CO_2_ concentration upon venting using outdoor air should converge to the global background concentration, which we assume to be 400 ppm. However, because ventilation may also be done against the air inside the school building we use the guidelines from Laussmann & Helm [43] and use the equation they derived for the temporal evolution of the CO_2_ concentration to determine the effective background concentration *C_a_* and the air change rate *λ* using a fit function of the form:

(1) $$C\left(t\right)=\left(C_{0}-C_{a}\right)\cdot e^{-\lambda \cdot t}+C_{a},$$

where *C*_0_ is the initial concentration upon start of the ventilation at *t* = 0 s. A nonlinear regression is applied to the measured raw data acquired in order to determine the background concentration. Based on the calculated air change rate *λ* we calculate the ventilation rate VR in L/s according to:

(2) VR= λ·V·13.6,

where *V* is the room volume in m³. The generation rate $V_{{\mathrm{CO}}_{2}}$ of CO_2_ inside rooms by occupants during class is calculated according to the model by Persily and de Jonge [44]:

(3) $$V_{{\mathrm{CO}}_{2}}=RQ\cdot BMR\cdot M\cdot \left(T/P\right)\cdot 0.000211,$$

which takes into account the respiratory quotient (RQ = 0.85 set as value here), the basal metabolic rate $BMR\;\mathrm{in}\;\frac{MJ}{day}$, the metabolic equivalent M, temperature T in K and Pressure *P* in kPa. With a known occupancy of each class the value of $V_{{\mathrm{CO}}_{2}}$ may be used to determine the physical activity of pupils, or vice versa. Apart from the CO_2_ concentration the thermal comfort is an important factor influencing the performance at school. Oftentimes, the temperature is used as the only parameter to assess the thermal comfort. However, humidity levels also significantly affect the thermal comfort. Although there are indications that children might experience thermal comfort differently than adults [45] we use the established ASHRAE Standard 55 to define thermal comfort [46] for people wearing winter clothing. Further large-scale measurement in various schools would be necessary to establish a reliable and more precise thermal comfort model for school children. For now we refer on ASHRAE 55, where good thermal comfort is achieved when temperatures are between 20 °C and 23 °C, and at the same time the relative humidity values are between 40 and 60% as described in [46]. Temperatures lower than 18 °C or higher than 25 °C as well as humidity levels below 20% or above 70% trigger a poor thermal comfort rating according to [47]. Intermediate values of temperature and humidity indicate mediocre but acceptable thermal comfort ratings. It should be taken into account that the thermal comfort evaluation has been made for the specific climatic locations in central Europe. In this case for the winter season and with a presumption of a typical winter indoor clothing level factor is set to 1.1 clo. This comfort function is easily adaptable to different climatic zones, clothing level and seasons of the year. In order to assign an overall indoor environmental quality score we include the CO_2_ concentration. We use the classification from [48] to evaluate the CO_2_ concentration levels: Levels below 1000 ppm indicate good air quality, values between 1000 and 2000 ppm medium quality, and values exceeding 2000 ppm poor air quality. Hence good indoor environmental quality requires both the thermal comfort as well as the air quality to be good and we assign three levels of IEQ to each room and at every time, which is indicated with green, yellow, and red shading in the graphs in line with previous research results [49,50]. 

## 3. Results and Discussion

**Figure 3:**
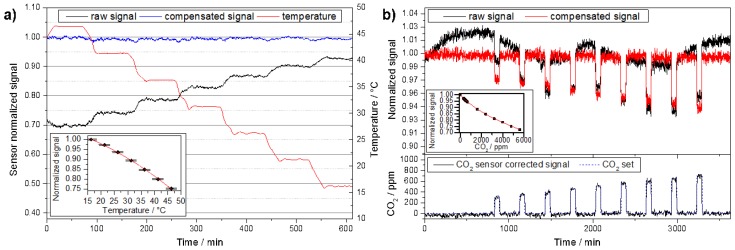
(**a**) Temperature calibration using a climatic chamber to enable corrections for changes in ambient temperature during operation of the sensor module. The blue curve shows the corrected signal. (**b**) Using a certified gas mixture the dependence of the sensor response on different CO_2_ concentrations has been established.

The raw data of the temperature calibration and the gas sensitive characterization of the CO_2_ module are shown in Figure 3. The photoacoustic amplitude excited by the LED in the absence of CO_2_ decreases with increasing temperature, mainly because the optical output the MID-IR decreases [29]. Increasing the temperature of the CO_2_ sensor module from 17 °C to 47 °C leads to a decrease in emitted power of approximately 32%. A parabolic regression for the sensor signal *S* of the form:

(4) $$S\left(T\right)=\;\sum_{i=0}^{2}\alpha_{i}T^{i},$$

with *T* the temperature in °C and $\alpha_{i}$ the regression parameters is applied to describe the dependence of the detector signal on the temperature. This function is achieved under normal operating conditions and takes into account self-heating of the LED at a duty cycle of 50% and the stated driving current of 80 mA. Changes in duty cycle or driving current would alter this function since this would impact on the LED emission. The inset of Figure 3a shows the results obtained with optimized values obtained via a Levenberg Marquart fit for which the $\alpha_{i}$ values read $\alpha_{0}$ = (1.07354 ± 0.01024), $\alpha_{1}$ = (−31.1 ± 7.5083) × 10^−4^ °C^−1^, and $\alpha_{2}$ = (−8.36047 × 10^−5^ ± 1.25458) × 10^−5^ °C^−2^, respectively.

After implementing this temperature correction a CO_2_ calibration is performed by determining the sensor response for different CO_2_ concentrations. Figure 3b shows the transient response of the gas sensor upon exposure of 9 different concentrations of CO_2_ in the range from 300–700 ppm in steps of 50 ppm. The inset shows the corresponding calibration curve that takes into account concentration values up to 5000 ppm. Using a cubic regression curve for the response *R* = *S*($c_{{\mathrm{CO}}_{2}}$ = 0 ppm)/*S*($c_{{\mathrm{CO}}_{2}}$) of the form:

(5) $$R\left(c_{{\mathrm{CO}}_{2}}\right)=\sum_{i=0}^{3}\beta_{i}\;{c_{{\mathrm{CO}}_{2}}}^{i},$$

with $c_{{\mathrm{CO}}_{2}}$ the CO_2_ concentration in ppm and $\beta_{i}$ the regression parameters yields: $\beta_{0}$ = 0.99742 ± 0.00197; $\beta_{1}$ = (−81.8975 ± 4.4699) × 10^−6^ ppm^−1^; $\beta_{2}$ = (9.50238 ± 1.92614) × 10^−9^ ppm^−2^; $\beta_{3}$ = (−6.38218 ± 2.26994) × 10^−13^ ppm^−^³. Characterization of the sensor type presented in previous work has showed no cross-sensitivities to humidity [30] and a detection range between 0–7000 ppm CO_2_ at 1 bar pressure [51] with operating temperatures between 15 °C and 50 °C (c.f. Figure 3). The operating principle prevents cross-sensitivities to gases that to not have absorption features that overlap with those of CO_2_. The lifespan of this type of sensor is determined by the lifetime of the LED light source, which we estimate to be at least 2 years, based on experimental data obtained previously [29]. The signal-to-noise ratio will worsen proportionally to the diminishing LED optical output [30] and hence the precision of the CO_2_ sensing module is affected accordingly. To counter this type of long-term effects a self-calibration routine may be implemented in the network by using prolonged periods without use of the school building, i.e., holidays, to automatically re-set the 400 ppm reading.

**Figure 4:**
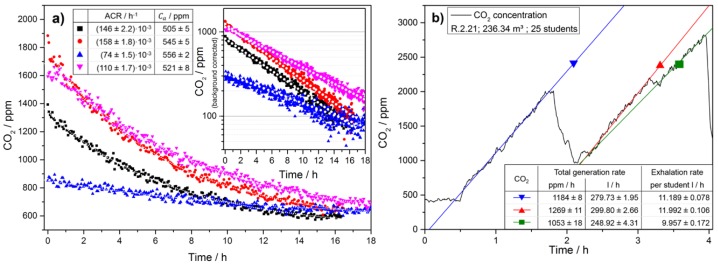
(**a**) CO_2_ decay curves measured at different times in the same classroom and the nonlinear regression curves used to estimate the background concentration (*C_a_*). The fit results are stated in the table in the inset. The inset on the right shows a logarithmic representation of the CO_2_ concentration values after subtraction of the background value *C_a_* to highlight the exponential decay behavior. (**b**) With closed class room doors the ventilation rate may be neglected as compared to the generation rate, which is why linear models apply. It also allows for determining the level of physical activity via the generation rate of CO_2_ during class. Knowing the volume of the room and the number of people inside allows for estimating the activity level, which may vary according to the specific activity performed in class.

After calibration all CO_2_ sensor modules and establishing the system at Remchingen school basic functions of the system such as ventilation rate and CO_2_ generation rate determination have been checked. Exemplary results for room R2.21 are shown in Figure 4 to highlight the procedure to determine *λ* and *C_a_* as well as the total CO_2_ generation rate. 

**Figure 5:**
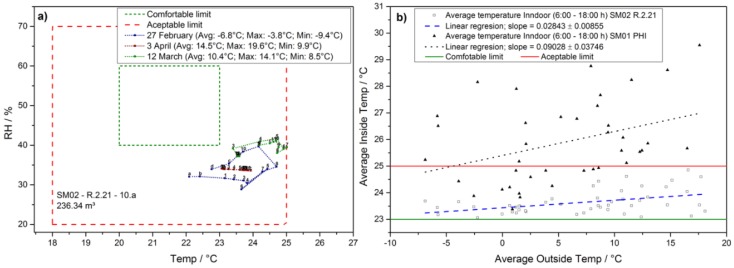
(**a**) Representation of the thermal comfort, taking into account temperature and relative humidity and its evolution during exemplary days, including a vacation day on 3 April 2018. Notably, good thermal comfort is never achieved because of too much heating during the winter season. (**b**) The correlation between inside and outside temperature are shown: A class room facing south (e.g., PHI) exceeds the acceptable limit of 25 °C in the majority of days, unlike class rooms facing north (e.g., R.2.21), due to more pronounced influence of heating by the sun.

The five rooms have been continuously monitored from 19 February 2018 until 10 April 2018. In Figure 5 we show the evolution of the thermal comfort during an exemplary day at the school in a class room with north orientation.

In this particular school, the heating system automatically starts to work at 5 am without active control with the intention to provide comfortable thermal conditions by 8 am. However, due to varying outdoor conditions this usually leads to an overheating early in the day. More precisely, on all days of the study the average room temperature during the morning has exceeded 23 °C, which is why a good thermal comfort range is only achieved in the early hours of each day. Additional heat generated by the class then quickly led to mediocre thermal comfort levels. While thermal comfort depends to a large extend on the subjective preference [6] and may still be judged acceptable by the pupils during most of the time the results show that an active thermal control taking into account outdoor temperature may lead to considerable cost savings via tailored heating. Moreover, the analysis of the data taking into account the orientation of each room shows that individual control of the thermal parameters taking into account radiative heating via sunshine is necessary to achieve good thermal comfort. 

The temperature data of the classroom summarizing the complete monitoring period is depicted in Figure 5b, where the average indoor temperature is plotted versus the average outside temperature on the particular day. The correlation between average outdoor temperature and average indoor temperature is more than three times stronger when comparing class rooms facing north with those facing south. A linear fit to the complete data set reveals a slope of s_S_ = (90 ± 40) mK/K for a room facing south and s_N_ = (28 ± 8) mK/K facing north. These results underline the necessity to individually monitor and control each room in a building according to its peculiarities. 

**Figure 6:**
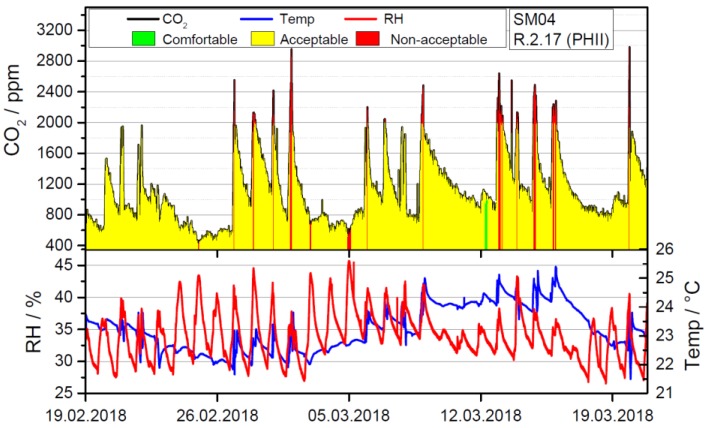
Transient evaluation of temperature, humidity and CO_2_ concentration in room R2.17 in the period between 19.02.2018 and 19.03.2018. Due to overheating the thermal comfort was only medium throughout the week. The CO_2_ levels have been recorded to be above 2000 ppm almost every day at some point.

Adding the information about the CO_2_ levels allows for a more comprehensive IEQ assessment and a transient, exemplary week is shown in Figure 6, using a color scheme to indicate overall IEQ.

**Figure 7:**
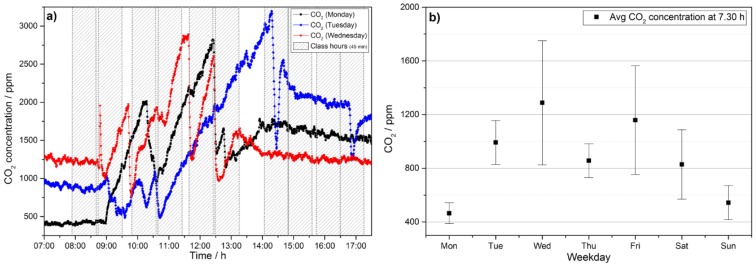
(**a**) Exemplary dynamics of the evolution of the CO_2_ level on three days of the week. Because doors and windows are closed after school finished CO_2_ can accumulate. (**b**) The accumulation leads to a constant increase in the background level of CO_2_, which makes it harder to maintain good CO_2_ levels the older the week gets.

Even though room R2.17 is the biggest room in this study in terms of volume and area it exceeds the recommended value of 1000 ppm after only about 22 min on average even when starting from the 400 ppm level. Because of noise pollution windows have to remain shut during a class and combining this with the high building standard leads to medium or poor indoor air quality even in a comparatively large room. The evolution of the CO_2_ level as the most important indicator for health and concentration capabilities has consequently been analyzed in more detail. To this end we have determined the background levels during the week as well as the evolution of the CO_2_ level within each day. In Figure 7 we present raw data and the analysis of the background concentration in the class at 7.30 am, i.e., before teacher or pupils enter the room. The transient raw data for Monday shows that CO_2_ levels quickly increase from background level of about 400 ppm CO_2_ to about 2000 ppm within 90 min. Afterwards the room is ventilated for 15 min, which leads to a drop of about 1000 ppm. Consequently, during the next class the CO_2_ level inevitably rises to almost 3000 ppm. School on that day has finished shortly before 2 pm and the doors are shut afterwards. Because of the high level of thermal insulation, the natural ventilation rate of the building is low, which leads to a high level of CO_2_ remaining in the class the next day, when classes start at about 1000 ppm background level in the first class given that day. Notably, the ventilation behavior plays a crucial role in maintaining CO_2_ concentrations at a reasonable level. In this regard, it is not only important to manage one class room well but because CO_2_ accumulated in the building as a whole, holistic strategies for CO_2_ management of the complete building are necessary. Even though the importance of ventilation is known in this school, the background level in class rooms not only increases during each day but also during the week. The average CO_2_ level in room R2.17 at 7.30 am is plotted in Figure 7b and shows that it is rising throughout the week. Thursday is an exception because the room is not used at all on Wednesdays, which leads to a drop in early morning CO_2_ levels consistent with natural ventilation.

**Figure 8:**
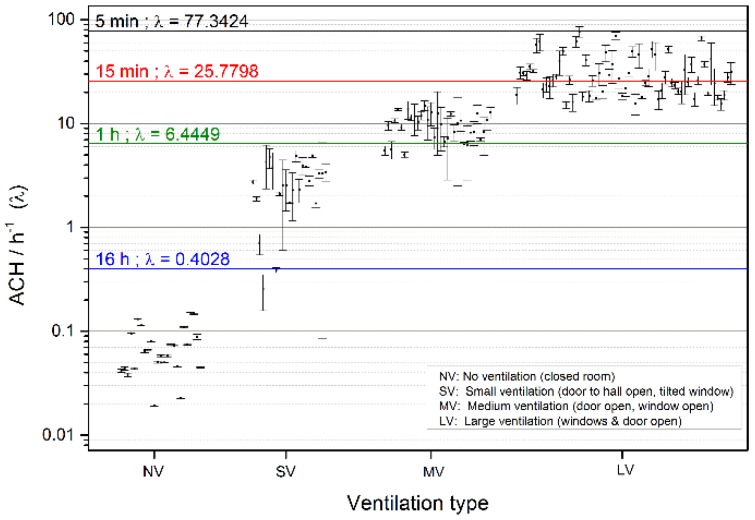
Air change rate as a function of the type of ventilation as well as the threshold ventilation rate required to achieve good IAQ. The results show that a 5 minute break is not sufficient to reestablish good IAQ. Only after 15 min of ideal and strong ventilation may low CO_2_ levels be achieved.

We have analyzed the different types of ventilation that are applied to the rooms and the ACR can be used to identify the type of action taken by the teacher and classify it. As expected the ACR greatly depends on opening the window and the air flow. The highest ACR results from opening several windows and doors simultaneously. This method is more than two orders of magnitude faster than the natural exchange of air of the room. Based on the results regarding the ventilation and CO_2_ background levels we have calculated which level of air change rate would be necessary to maintain CO_2_ levels consistent with good IAQ for ventilation times of 5 min, 15 min, 1 h, and 16 h respectively. The results are plotted in Figure 8 and show that natural ventilation of the building is insufficient to achieve good IAQ even after 16 h. 

Even more attention-grabbing, the determined average natural ventilation rate of the building is below 0.1, which means that even a weekend is not long enough to recuperate background levels of 400 ppm CO_2_ if teachers fail to vent the room on Friday. This ultimately leads to an accumulation of CO_2_ during weeks and months, if no appropriate action is taken. Also a short 5 min break in between classes is insufficient to obtain good IAQ. Only a 15 min break and maximal ventilation would allow for good IAQ after a class. As a consequence even in schools with teachers that are highly aware of IEQ issues, such as at Remchingen Gymnasium, it is highly improbable that good IEQ can be achieved at all without the help of indoor air quality indicating systems.

## 4. Conclusions

High building standards and good thermal insulation have led to considerable saving in energy expenditure of buildings. Here we have shown that the strong suppression of the natural ventilation of buildings makes it very difficult to maintain good IAQ without active HVAC systems. CO_2_ not only accumulates during each school day but also during the course of the week leading to ever poorer IAQ. Only with ideal ventilation after each class is it possible to maintain good IAQ. At the same time schools are overheated to varying degrees depending on the orientation of the room. The results also show that it is important to monitor and control rooms individually to design mitigation strategies tailored to each room. This does not take away from the fact that a building wide strategy for IAQ control is necessary if good air quality is to be achieved. Because most schools are not equipped with active environmental control systems and thermal insulation is an important cornerstone for a sustainable energy landscape, the monitoring and implementation of intelligent ventilation strategies is much in need. In this regard, wireless sensor networks are a suitable technique to provide data on various temporal and spatial scales. The limiting factor is oftentimes the cost of each individual sensing node and here we have showed that low-cost, reliable, and selective gas sensing nodes are indeed feasible.
